# Engineering the *Saccharomyces cerevisiae* β-Oxidation Pathway to Increase Medium Chain Fatty Acid Production as Potential Biofuel

**DOI:** 10.1371/journal.pone.0084853

**Published:** 2014-01-21

**Authors:** Liwei Chen, Jianhua Zhang, Wei Ning Chen

**Affiliations:** School of Chemical and Biomedical Engineering, College of Engineering, Nanyang Technological University, Singapore, Singapore; Oak Ridge National Laboratory, United States of America

## Abstract

Fatty acid-derived biofuels and biochemicals can be produced in microbes using β-oxidation pathway engineering. In this study, the β-oxidation pathway of *Saccharomyces cerevisiae* was engineered to accumulate a higher ratio of medium chain fatty acids (MCFAs) when cells were grown on fatty acid-rich feedstock. For this purpose, the haploid deletion strain *Δpox1* was obtained, in which the sole acyl-CoA oxidase encoded by *POX1* was deleted. Next, the *POX2* gene from *Yarrowia lipolytica*, which encodes an acyl-CoA oxidase with a preference for long chain acyl-CoAs, was expressed in the *Δpox1* strain. The resulting *Δpox1* [*pox2+*] strain exhibited a growth defect because the β-oxidation pathway was blocked in peroxisomes. To unblock the β-oxidation pathway, the gene *CROT*, which encodes carnitine O-octanoyltransferase, was expressed in the Δ*pox1* [*pox2+*] strain to transport the accumulated medium chain acyl-coAs out of the peroxisomes. The obtained Δ*pox1* [*pox2+, crot+*] strain grew at a normal rate. The effect of these genetic modifications on fatty acid accumulation and profile was investigated when the strains were grown on oleic acids-containing medium. It was determined that the engineered strains Δ*pox1* [*pox2+*] and Δ*pox1* [*pox2+, crot+*] had increased fatty acid accumulation and an increased ratio of MCFAs. Compared to the wild-type (WT) strain, the total fatty acid production of the strains Δ*pox1* [*pox2+*] and Δ*pox1* [*pox2+, crot+*] were increased 29.5% and 15.6%, respectively. The intracellular level of MCFAs in Δ*pox1* [*pox2+*] and Δ*pox1* [*pox2+, crot+*] increased 2.26- and 1.87-fold compared to the WT strain, respectively. In addition, MCFAs in the culture medium increased 3.29-fold and 3.34-fold compared to the WT strain. These results suggested that fatty acids with an increased MCFAs ratio accumulate in the engineered strains with a modified β-oxidation pathway. Our approach exhibits great potential for transforming low value fatty acid-rich feedstock into high value fatty acid-derived products.

## Introduction

The renewable synthesis of fatty acid-derived biofuels and chemicals has gained considerable attention in recent years. In several studies, fatty acids are overproduced in microbes and later converted to fuels or chemicals through chemical conversion [1] or bioconversion [2], [3]. Other studies directly utilize fatty acid-rich feedstock as a carbon source to produce biofuels and chemicals through the complete oxidation of fatty acids [4] or using the intermediates of β-oxidation [5], [6]. The availability of fatty acids from nonedible fatty acid-rich crops, oleaginous fungi/algae and industrial by-products encourage the use of fatty acid feedstock as a cheap carbon source. In addition, fatty acids have a product yield advantage over sugars when used as a carbon source due to the efficient β-oxidation metabolism process [4], which has 100% carbon recovery. In short, fatty acids can be exploited as an alternative feedstock to lignocellulosic sugars for microbial bioconversion platforms.

*Saccharomyces cerevisiae* has been commonly used in industrial fermentation. It has better tolerance to organic solvent than Escherichia *coli* and also owns a well-studied β-oxidation pathway. Aiming to utilize fatty acid-rich feedstock to produce biofuels or biochemicals, we investigated fatty acid metabolism in *S. cerevisiae*. We intended to generate more medium chain fatty acids (MCFAs), which are of higher value compared to other fatty acids [7]. Because fatty acid-rich feedstock usually contains sugars and fatty acids in carbon length at C16 and C18 [8], we used oleic acid as a glucose co-substrate to test the bioconversion ability of engineered strains during culture.

Fatty acid β-oxidation is the main pathway for fatty acid degradation in yeast. It enables yeast cells to grow on medium containing fatty acids as the sole carbon and energy source. This process is carried out in the peroxisomes of *S. cerevisiae* [9], [10]. The first and rate-limiting step is catalyzed by acyl-CoA oxidase using acyl-CoA as the initial substrate. Before the degradation process begins, long chain fatty acids (LCFAs) are activated into long chain acyl-CoAs by acyl-CoA synthetases in the cytosol and then are transported into peroxisomes by Pxa1p/Pxa2p. In contrast to LCFAs, MCFAs can freely enter peroxisomes and become medium chain fatty acyl-CoAs with the help of acyl-CoA synthetases. In *S. cerevisiae*, the *POX1* gene encodes the only acyl-CoA oxidase, which can oxidize short to long chain acyl-CoAs. Disruption of *POX1* prevents yeast survival on medium using oleic acid as the sole carbon source [11]. In contrast to *S. cerevisiae*, the yeast *Yarrowia lipolytica* (*Y. lipolytica*) has several acyl-CoA oxidases with different chain length specificities [12], [13]. It has been demonstrated that modification of *POX* genotype can affect β-oxidation and further influence lipid accumulation in *Y. lipolytica* [14], but there is no data about changes specifically to MCFAs.

**Figure 1:**
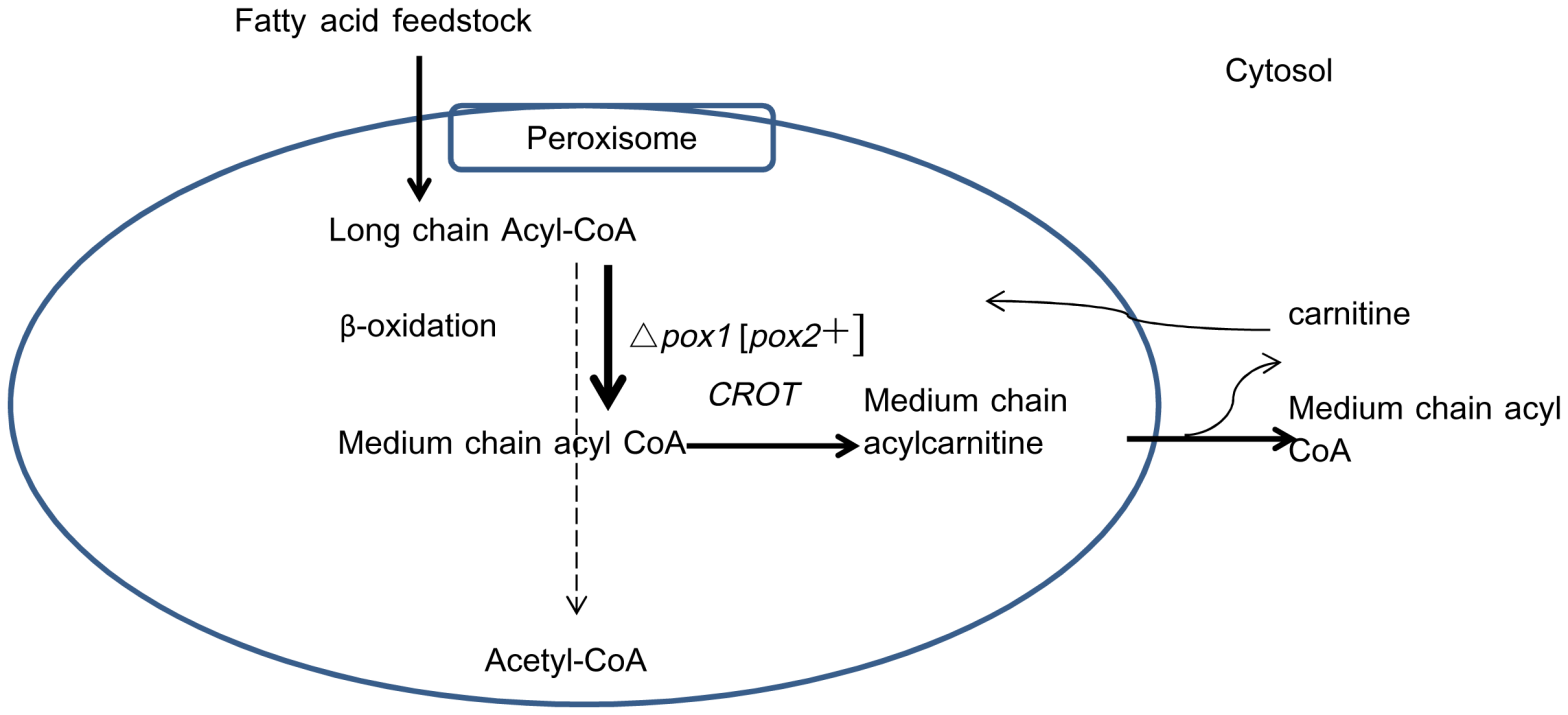
Genetic modification of the β-oxidation pathway in *Saccharomyces cerevisiae*. The dashed line represents the original pathway; the solid line represents the modified pathway. The only acyl-CoA oxidase (encoded by the gene *POX1*) in the *S. cerevisiae* genome was deleted, and the *POX2* gene from *Yarrowia lipolytica*, which encodes acyl-CoA oxidase with a preference for long chain acyl-CoAs, was expressed. To unblock the β-oxidation pathway, peroxisomal carnitine octanoyltransferase (*CROT*) from *Mus musculus* was also expressed to transport medium chain fatty acyl-CoAs out of peroxisomes.

To use fatty acid-rich feedstock to generate tailored fatty acids for biofuel production, we modified the β-oxidation pathway of *S. cerevisiae*, both to maintain LCFA oxidation and to prevent MCFA oxidation. The strain was engineered with the intention to accumulate more fatty acids with a higher ratio of MCFAs when cells were grown on fatty acid-rich feedstock. To achieve this aim, the *S. cerevisiae* deletion strain *Δpox1* was obtained in which the *POX1* gene, the sole acyl-CoA oxidase, was deleted. Then, acyl-CoA oxidase Aox2p (encoded by *POX2*) from *Y. lipolytica* [15], which has a preference for long-chain acyl-CoA substrates, was expressed. The replacement of *POX1* with *POX2* led to the blocking of β-oxidation pathway. To unblock the β-oxidation pathway in the Aox2p-expressing strain, peroxisomal carnitine octanoyltransferase (*CROT*) [16] from *Mus musculus* was also expressed. This transferase moves medium chain fatty acyl-CoAs out of peroxisomes by producing medium chain fatty acyl carnitine esters. The modified pathway is depicted in Figure 1. The effect of β-oxidation cycle modification on fatty acid profile and accumulation was investigated and discussed.

## Materials and Methods

### Yeast strains and growth conditions

The yeast strains used in this study were derived from *S. cerevisiae* BY4741 (Mat a; his3Δ1; leu2Δ0; met15Δ0; ura3Δ0, Acc. noY00000). The WT strain BY4741, the *Δpox1* deletion strain (BY4741; Mat a; his3Δ1; leu2Δ0; met15Δ0; ura3Δ0; YGL205w::kanMX4, acc. no Y04571) and the plasmid pVTU260 were obtained from EUROSCARF (Germany). The strains were cultivated at 30°C in YPD complete medium [1% Bacto yeast extract, 2% Bacto peptone, 2% dextrose], supplemented with 200 µg/ml G418 (Sigma, Singapore) when necessary. SC Minimal Medium (YNBD) was comprised of 0.67% yeast nitrogen base (without amino acids but with ammonium sulfate, Invitrogen), 2% dextrose and amino acid drop out (without URA, Clontech). YNBD_0.5_O_3_ (YNB supplemented with 0.5% dextrose and 3% oleic acid (from Fluka 75096)) was used for the optimum accumulation of fatty acids. Minimal oleic acid medium (YNBO) was used to test the fatty acid degradation ability of the engineered strains. YNBO was YNB supplemented with 1% oleic acid as the sole carbon source.

### Construction of plasmids pVTU260-POX2 and pVTU260-POX2-CROT

Molecular cloning was performed according to standard procedures. The QIAprep Spin Miniprep Kit was used for plasmid preparation. The QIAquick Gel Extraction Kit and QIAquick PCR Purification Kit (QIAGEN) were used for purification of gene fragments.

The gene *POX2* (GenBank: accession no. CR382132.1) encoding acyl-CoA oxidase 2 (Aox2p) was synthesized by Geneart (American) with optimized codons. The GenBank accession number for the codon-optimized sequence of *POX2* is KC912711. The primers used for PCR were POX2F-pvtu260 (5′ CGGCTAGCCGCAAAATGAATCCAAACAATAC) and POX2R-pvtu260 (5′ CGGGATCCGGTCAATGGTGATGGTGATGATGTTCTTCATCCAATTC). The forward primer introduced a unique *Nhe*I restriction site and a yeast consensus sequence (CAAA) before the 5′ initiation codon. The reverse primer introduced a His-tag sequence before the 3′ termination codon and a unique *Bam*HI restriction site after the termination codon. The PCR products were digested with *Nhe*I and *Bam*HI and then ligated into the pVTU260 vector, which had also been digested with *Nhe*I and *Bam*HI. The ligation product was transformed into *E. coli* Top10. The resulting plasmid pVTU260-POX2 was verified by restriction analysis and sequencing.

The gene *CROT* (NM_023733.3) was also codon optimized and synthesized by Geneart. The GenBank accession number for the codon-optimized sequence of *CROT* is KC912712. Codon-optimized CROT was amplified using the primers TEFp-CROTF (5′ GTAGGAGGGCTTTTGTAGATGCTAGCCAAAGGTAAGCCTATCCCTAACCCTCTCCTCGGTCTCGATTCTACGATGGA) and CROTR-TEFt (5′ ACAACACTCCCTTCGTGCTTCCCCCCGGGGGGTCACAAGTGGGCGGTATTCATC). An *Nhe*I restriction site, yeast consensus sequence and V5-tag was introduced before the 5′ initiation codon. Because Crot contains a peroxisome-targeting signal peptide (AHL) at the C-terminal end, the V5-tag was placed at the N-terminal to prevent interference with the peroxisome-targeting signal peptide. The *Ashbya gossypii TEF2* promoter and terminator were PCR-amplified individually from pUG27 [17] using the primer pair TEFp-pUG27-F 5′ GAAGATCGTCGTTTTGCCAGGTGACCGACAACCCTTAATATA and TEFp-R 5′ GGCTTACCTTTGGCTAGCATCTACAAAAGCCCTCCTAC and the primer pair TEFt-F 5′ CCGCCCACTTGTGACCCCCCGGGGGGAAGCACGAAGGGAGTGTTGT and TEFt-R 5′ TTAATTATATCAGTTATTACCCGGGTGAGCGAGGAAGCGGAAGA. The *CROT* gene expression cassette containing the three PCR fragments was assembled into pVTU260-POX2 digested with *Bst*EII and *Xma*I using the Cold Fusion Cloning Kit (SBI). The resulting plasmid pVTU260-POX2-CROT was verified through restriction analysis and sequencing.

### Cell transformation and control cell development

The plasmids pVTU260-POX2 and pVTU260-POX2-CROT were transformed separately into Δ*pox1* yeast competent cells using the lithium acetate method [18]. Control cells were created by transforming the empty vector pVTU260 into the WT and Δ*pox1* strain. To examine if expression of Aox2p caused growth defects, pVTU260-POX2 was also transformed into the WT strain as a control. Colonies on the screening plates were verified by colony PCR.

### Western blot analysis

After 24 h, 5 ml of cell culture was collected by centrifugation at 8000 rpm. After washing once with lysis buffer (50 mM HEPES, 5% glycerol, 1 mM DTT, 1 mM PMSF, 1 mM EDTA), the pellet was resuspended in lysis buffer to an OD_600_ of 100. The peroxisome proteins were isolated using a peroxisome isolation kit (Sigma). Total proteins were mixed with loading buffer and boiled at 95°C for 5 min and separated on a 10% SDS%-PAGE (Bio-Rad). The separated proteins were transferred to a nitrocellulose (NC) membrane (GE Hybond, USA) at 21 V for 1 h 20 min using a semi-dry apparatus. After incubating the NC membrane in blocking buffer (PBST+5% nonfat, dry milk, w/v) for 1 hour at room temperature, the membrane was probed with an anti-V5 antibody or an anti-His antibody (Sigma) at 4°C overnight. Then, the membrane was incubated with an anti-mouse IgG HRP-conjugated secondary antibody (#31430, Pierce) at the dilution suggested by the manufacturer. After washing with PBST buffer, the signal was detected with the ECL Western blot reagent.

### Growth tests of control and engineered strains

Yeast cells were collected after overnight culture in liquid YNBD medium at 30°C. The cells were resuspended at a cell density of 5×10^3^ cells/µl after washing twice in sterile distilled H_2_O. Cells (5 µl) were spotted onto YNBD or YNBO-agar plates. The first drop contained 2.5×10^4^ cells, and each subsequent drop was diluted six fold more than the previous one.

### Sample preparation for metabolite and fatty acid analysis

Five milliliters of liquid YNBD medium was inoculated with a single colony from agar plate and cultured overnight at 30°C with shaking. Then, the overnight culture was used to inoculate 50 ml liquid YNBD or YNBD_0.5_O_3_ medium. The initial OD_600_ was adjusted to approximately 0.3. The cells were cultured by shaking at 250 rpm and 30°C. Same amounts of yeast cells (8×10^7^ cells) were separately collected at different growth time points. The intracellular metabolites were extracted from control and engineered cells based on previously published work [19], [20]. In general, the culture sample was centrifuged for 5 min at 4°C. The supernatant was collected for extracellular metabolite analysis. The cell pellet was washed twice with cold methanol (<−40°C) and then collected.

### Fatty acid analysis of cells and culture supernatants

The lipids were extracted from yeast cells using an adjusted chloroform-methanol 2∶1 method [21]. The cell pellet was resuspended in 1000 µl of 0.9% NaCl solution and then acidified with 200 µl of acetic acid. As an internal standard (IS) to correct for metabolite loss during sample preparation, 10 µl of 10 mg/ml heptadecanoic acid and heptanoic acid dissolved in ethanol was added to the extraction solvent. After adding an equal volume of glass beads, the cells were disrupted in a FastPrep®-24 Instrument (6004-500, MP Biomedicals) for 30 s and cooled in ice for 30 s; this procedure was repeated 4 times. Then, 3 ml of a chloroform-methanol 2∶1 mixture was added, and the samples were inverted several times, vortexed vigorously, and centrifuged at 10000 g for 10 min at 4°C. The aqueous layer and cell debris were transferred to a new tube using aspiration. The chloroform layer (lower) was collected. An additional 3 ml of the chloroform-methanol 2∶1 mixture was added to the aqueous layer to further extract lipids. Then, the chloroform layer was rotary evaporated to near dryness overnight using a TurboVapH LV Concentration Workstation.

For the fatty acids in the culture medium, the Solid-Phase Extraction (SPE) method [22] was adopted. First, the SPE column (Strata C18-E 200 mg/3 ml) was activated with 2.5 ml methanol and washed with 5 ml water. Then, 10 ml culture supernatant to which 0.5 ml 1 M HCl, 2 µl 10 mg/ml heptadecanoic acid and 2 µl 10 mg/ml heptanoic acid were added was passed through the SPE column. The SPE column was washed with 5 ml water and dried at room temperature. Next, 1.5 ml chloroform was used for fatty acid elution. The chloroform in elution products was also evaporated to near dryness.

Fatty acid analysis was performed according to previous work [22]. Fatty acid methyl esters (FAMEs) mix C_8_–C_24_ (SUPELCO, 18918) was used as a standard. Three independent experiments were conducted. The dried lipid residue was redissolved in 500 µl BF3-methanol 10% (FLUKA, 15716) and incubated in a sealed vial in a 95°C heater for 20 min. FAMEs were extracted with 300 µl n-hexane after the addition of 300 µl saturated NaCl in water. All of the samples were analyzed using an Agilent Technologies 7890A-5975C GC-MS system equipped with a HP-5MS capillary column (30 m×0.250 mm i.d.; film thickness: 0.25 µm; Agilent J&W Scientific, Folsom, CA, USA). Helium was used as a carrier gas at 1.1 mL/min. The inlets and MS source temperatures were maintained at 250 and 230°C, respectively. For fatty acid analysis, the oven temperature was maintained at 80°C for 1 min and ramped to 250°C at a rate of 7°C.min^−1^, then held at 250°C for 10 min. Data were acquired in a full scan from 35 to 600 m/z. The detected FAME peaks were integrated. Amounts were calculated with reference to the IS heptadecanoic acid, and the relative response factors were calculated.

### Metabolic profiling

The sample for metabolic profiling was derivatized according to previous work [23]. For metabolic profiling, the same GC-MS system was used. The oven temperature was maintained at 75°C for 4 min and raised at 4°C.min^−1^ to a final temperature of 280°C and held for 2 min. Mass spectra were recorded from 35 to 600 m/z with a scan time of 0.2 s. Chromatogram acquisition and mass spectra identification were processed using the Agilent MSD Chemstation Data Analysis software. Chemical identification of the detected metabolite peaks was performed by searching the NIST08 mass spectral library. The compounds were quantified from the peak area relative to the IS ribitol. No response factors were made.

## Results

### Gene modification of the β-oxidation pathway in *S. cerevisiae*

**Figure 2:**
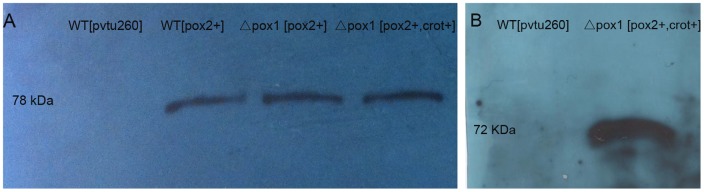
Western for Aoxp2 and Crot. A. Expression of Aoxp2 in WT [*pox2+*], Δ*pox1* [*pox2+*] and Δ*pox1* [*pox2+, crot+*]. B. Expression of Crot in Δ*pox1* [*pox2+, crot+*].

The modification of the β-oxidation pathway is based on the deletion of one gene and the expression of two proteins. The deletion strain Δ*pox1* was obtained from EUROSCARF and tested for growth on YNBO. The Δ*pox1* strain was unable to grow on medium in which oleic acid was the only carbon source. This is consistent with a previous report [11]. To express acyl-CoA oxidase 2 (Aox2p), which has a preference for long chain substrates, the multi-copy plasmid pVTU260 was used. The plasmid pVTU260-POX2 encoding the *POX2* gene under the control of the constitutive promoter *ADH1* was transformed into Δ*pox1*, resulting in the strain Δ*pox1* [*pox2+*]. To express carnitine O-octanoyltransferase, the gene *CROT* was cloned into pVTU260-POX2 under the control of the constitutive promoter *TEF*. The resulting plasmid pVTU260-POX2-CROT was transformed into strain Δ*pox1*, creating strain Δ*pox1* [*pox2+, crot+*]. These two enzymes were successfully expressed in the Δ*pox1* strain. Western results are shown in Figure 2. Aox2p is approximately 78 kDa; Crot is approximately 72 kDa.

### Growth phenotype analysis

**Figure 3:**
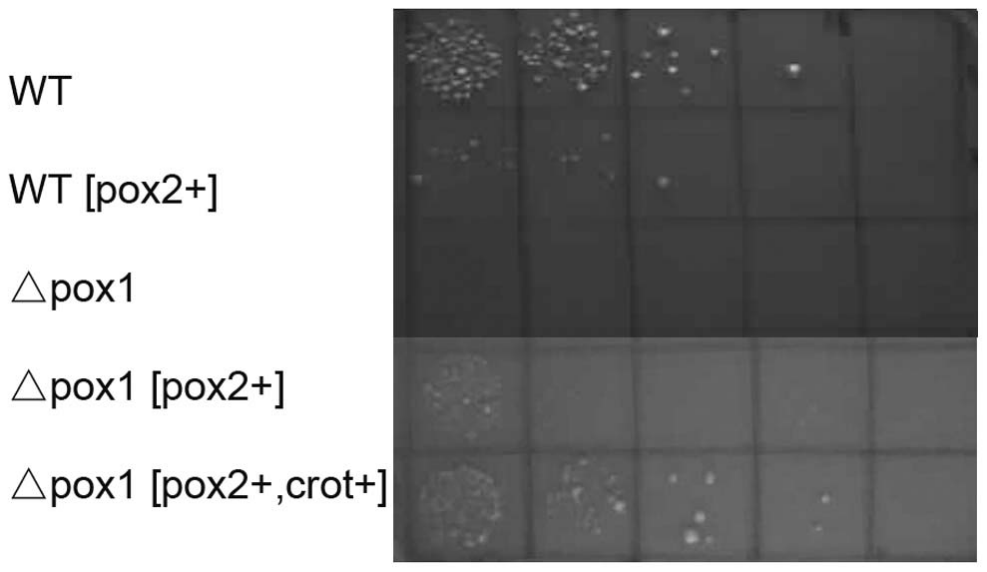
Growth tests on the engineered strains and the WT strain in YNBO medium. WT and Δ*pox1* transformed with the empty vector pvtu260, WT transformed with pvtu260-pox2, Δ*pox1* transformed with pvtu260-pox2 and pvtu260-pox2-crot were all cultured overnight in liquid YNBD medium at 30°C. Yeast pellets were collected and resuspended at a cell density of 5×10^3^ cells/µl after being washed twice in sterile distilled H_2_O. To test growth, 5 µl were spotted on YNBO-agar plates. The first drop contained 2.5×10^4^ cells, and each subsequent drop was diluted six fold more than the previous one.

**Figure 4:**
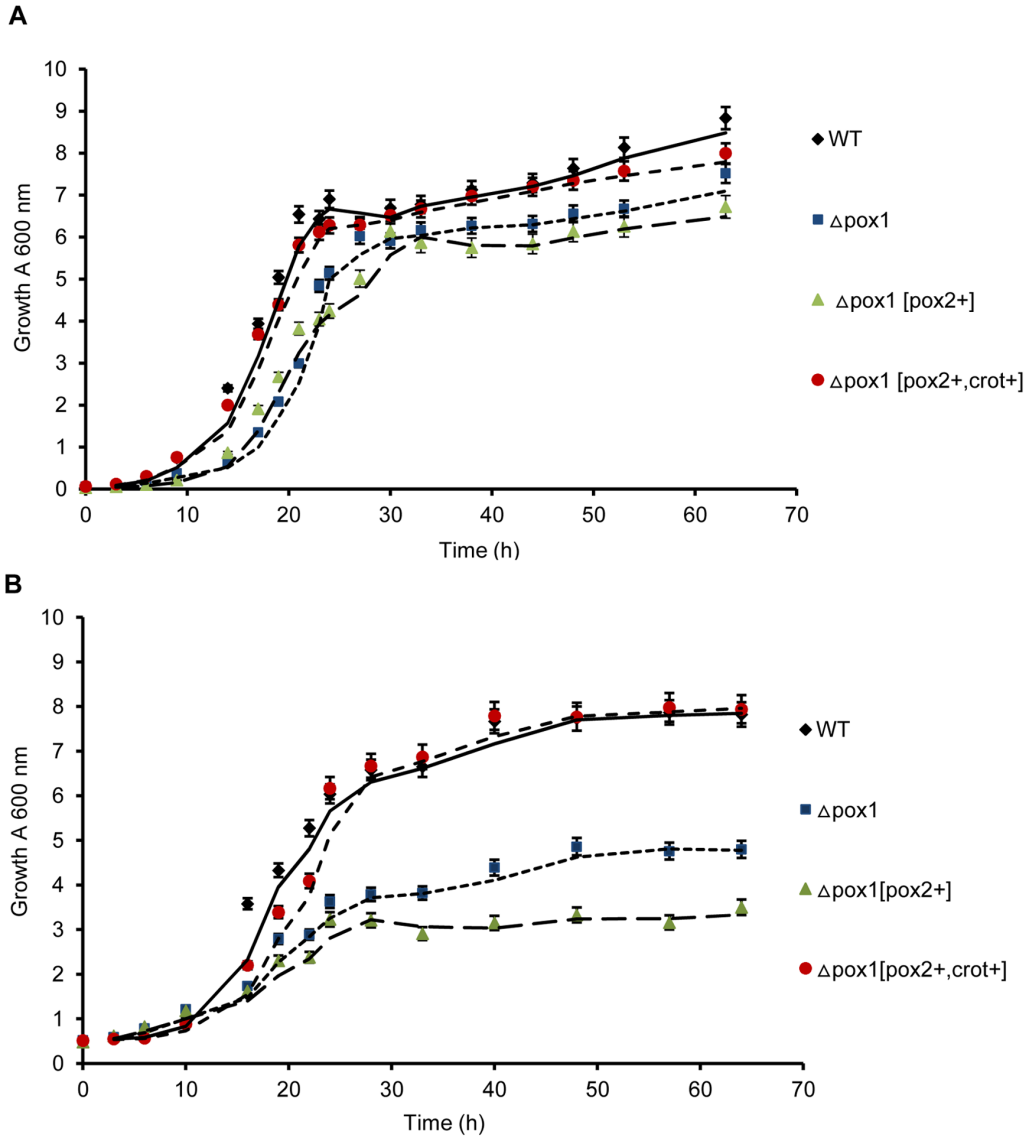
Growth curves of the engineered strains and the WT strain. A. Growth curves of the engineered strains and the WT strain in YNBD, B. Growth curves of the engineered strains and the WT strain in YNBD_0.5_O_3_. The results are the mean values of three independent experiments.

Growth tests were performed to evaluate the potential negative effects of the genetic modification on the growth of yeast cells. The Δ*pox1* strain did not grow on the oleic acid-based (YNBO) agar plate. The Δ*pox1* [*pox2+*] and WT [*pox2+*] strains showed a severe growth defect (Figure 3), whereas Δ*pox1* [*pox2+, crot+*] grew similar to the WT. When all the strains were cultured in glucose-based (YNBD) medium (as shown in Figure 4A), there was no severe growth defect observed in the engineered strains. It was observed that Δ*pox1* [*pox2*+] grew more slowly than the WT strain. Δ*pox1* [*pox2+, crot+*] showed a growth rate similar to the WT strain. However, in oleic acid-glucose-based (YNBD_0.5_O_3_) medium (Figure 4B), Δ*pox1* showed reduced growth compared to the WT strain. The growth of Δ*pox1* [*pox2*+], expressing Aox2p, was more severely affected than the Δ*pox1* strain. The Δ*pox1* [*pox2+, crot+*] strain did not show a growth defect. The Δ*pox1* growth defect in oleic acid-containing medium was due to defective fatty acid degradation. Oleic acid could not be utilized by Δ*pox1* as a carbon source. The growth defect in strain Δ*pox1* [*pox2*+] indicated that β-oxidation was not fully rescued by Aox2p. Because Aox2p has very low activity towards acyl-CoAs with fewer than ten carbons [14], the over-accumulation of medium chain acyl-CoAs from LCFA degradation in the peroxisome prevented further fatty acid oxidation, which restricted the carbon flux toward β-oxidation. The defective growth of the WT [*pox2+*] strain further supported this conclusion. In strain Δ*pox1* [*pox2+, crot+*], the medium chain acyl-CoAs accumulated were transferred to carnitine by Crot [24] and subsequently removed from peroxisomes, allowing unobstructed carbon flux.

### Analysis of fatty acid composition in cell extracts and culture supernatants

To examine the effect of Aox2p and Crot on fatty acid accumulation and composition, the intracellular and extracellular fatty acids of cultured cells at the stationary phase were analyzed. When the engineered strains were cultured in YNBD medium, the total fatty acids in the engineered strains increased less than 10% compared with the WT strain. There was not an obvious change in the fatty acid profile and C8:0 and C10:0 were not detected in the culture supernatant of any of the engineered strains (data not shown).

**Figure 5:**
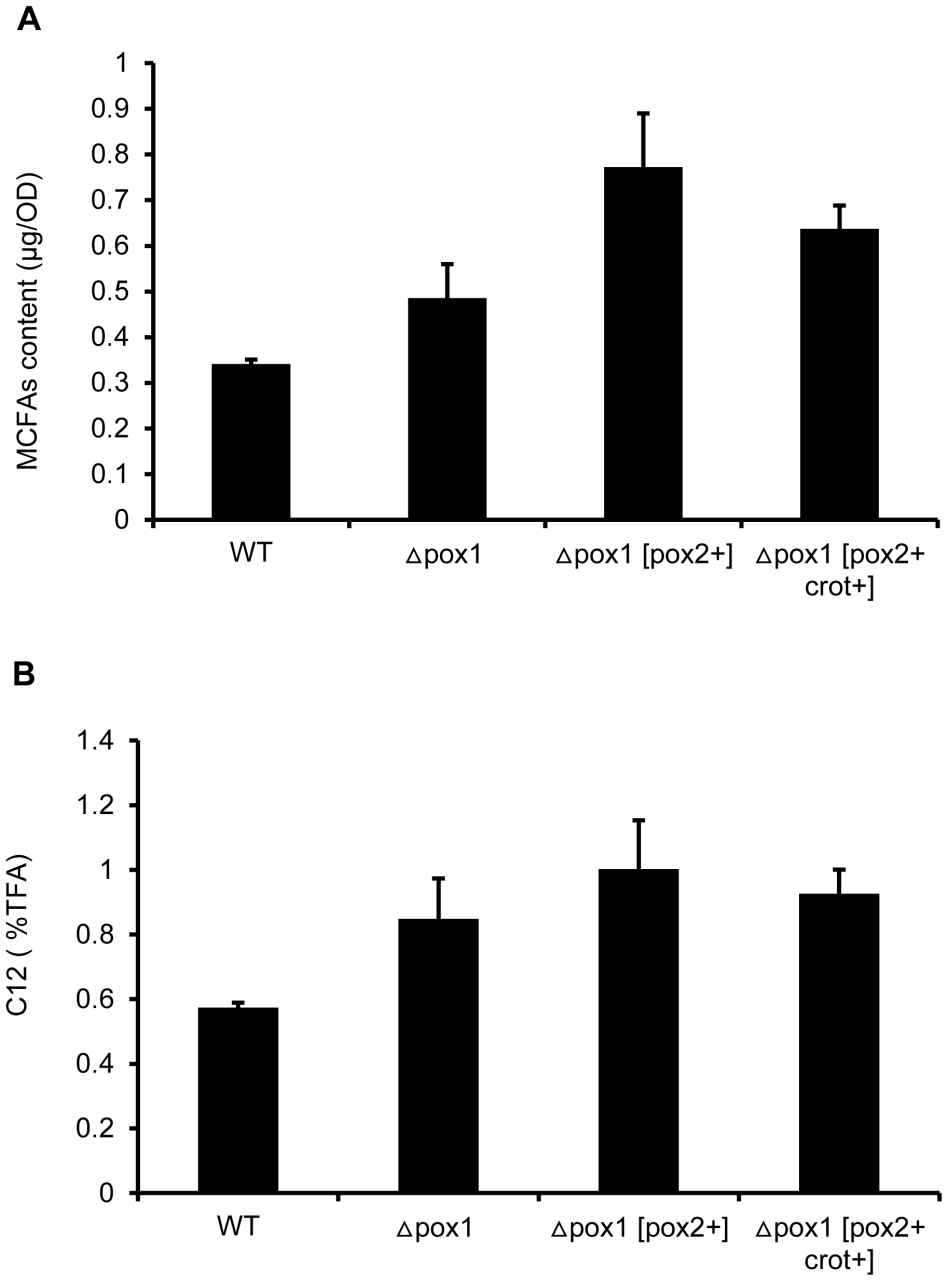
MCFAs analysis of the cell extracts. A. MCFAs content in the cell extracts from the engineered strains and the WT strain after 24_0.5_O_3_ medium. B. Composition of MCFAs from the total fatty acids in the cell extracts from the engineered strains and the WT strain at 24 h when cultured in YNBD_0.5_O_3_ medium. Cells were collected after 24 h of growth in YNBD_0.5_O_3_ medium, and the cell pellet was separated by centrifugation. The total fatty acids were extracted and detected. There was C12:0 but not C8:0 and C10:0 in the cell extract, so the MCFAs were only C12:0 in this case.

When cells were cultured in YNBD_0.5_O_3_, the fatty acid accumulation and composition of the engineered strains were different from WT strain. For intracellular fatty acids from the cell extract at stationary phase (Table 1 and Table 2), the total fatty acids in strains Δ*pox1* [*pox2*+] and Δ*pox1* [*pox2*+, *crot*+] increased 29.5% and 15.6% compared to the WT strain, respectively. There is no increase of total fatty acids in the Δ*pox1* strain. The unsaturated fatty acids ratio (unsaturated fatty acids compared to saturated fatty acids) increased to 0.083 (Δ*pox1*), 0.073 (Δ*pox1* [*pox2*+]) and 0.078 (Δ*pox1* [*pox2*+, *crot*+]) compared to 0.041 (WT). In terms of MCFAs, there was an increase in MCFA content (present only as lauric acid in the cell extract) in the strains Δ*pox1*, Δ*pox1* [*pox2*+] and Δ*pox1* [*pox2*+, *crot*+] compared with the WT strain. Lauric acid (C12:0) content in Δ*pox1* (0.486 µg/OD), Δ*pox1* (*pox2*+) (0.77 µg/OD) and Δ*pox1* [*pox2*+, *crot*+] (0.64 µg/OD) were increased 1.42-, 2.26- and 1.87-fold compared to the WT strain (0.34 µg/OD) (Figure 5A). In the strains Δ*pox1*, Δ*pox1* [*pox2*+] and Δ*pox1* [*pox2*+, *crot*+], lauric acid constituted 0.85%, 1.0% and 0.92% of the total fatty acids (Figure 5B) compared to 0.57% in the WT, which corresponds to a 1.47-, 1.75- and 1.61-fold increase. The data demonstrated that modification of the β-oxidation pathway changed the total fatty acids, unsaturated fatty acids/saturated fatty acids ratio and the MCFAs ratio in yeast cells.

**Table 1:** Fatty acid production in the cell extract of the WT and the engineered strains^a^.

Fatty acid type	WT	Δ*pox1*	Δ*pox1* [*pox2+*]	Δ*pox1*[*pox2+ crot+*]
C12:0	0.341	0.486	0.772	0.637
C14:0	1.183	1.590	2.143	1.855
C16:1	0.881	1.384	1.878	1.788
C16:0	30.623	27.675	35.794	32.535
C18:1	1.449	2.977	3.334	3.170
C18:0	24.889	22.959	32.890	28.548
C20:0	0.120	0.143	0.202	0.227
Total fatty acids	59.486	57.214	77.014	68.760

^a^ Data represent fatty acid composition in µg/OD cell when WT (wild-type) and engineered strains were cultured in YNBD_0.5_O_3_ medium. The values are the means from three experiments examining the cell extracts at 24 h. The standard deviations were <5% of the values.

**Table 2:** Fatty acid composition changes in cell extract comparing the WT and the engineered strains in YNBD_0.5_O_3_ medium^b^.

Fold changes of fatty acids	Δ*pox1*	Δ*pox1* [*pox2+*]	Δ*pox1*[*pox2+ crot+*]
C12:0	1.422	2.262	1.866
C14:0	1.344	1.812	1.568
C16:1	1.570	2.132	2.029
C16:0	0.904	1.169	1.062
C18:1	2.054	2.300	2.187
C18:0	0.923	1.322	1.147
C20:0	1.194	1.681	1.889
Total fatty acids	0.962	1.29	1.156

^b^ The fold change data for the engineered strains were analyzed compared to the WT (wild-type) strain. The amount of fatty acids in the WT strain was set as 1. The values are the means from three experiments examining cell extracts at 24 h. The standard deviations were <5% of the values.

**Figure 6:**
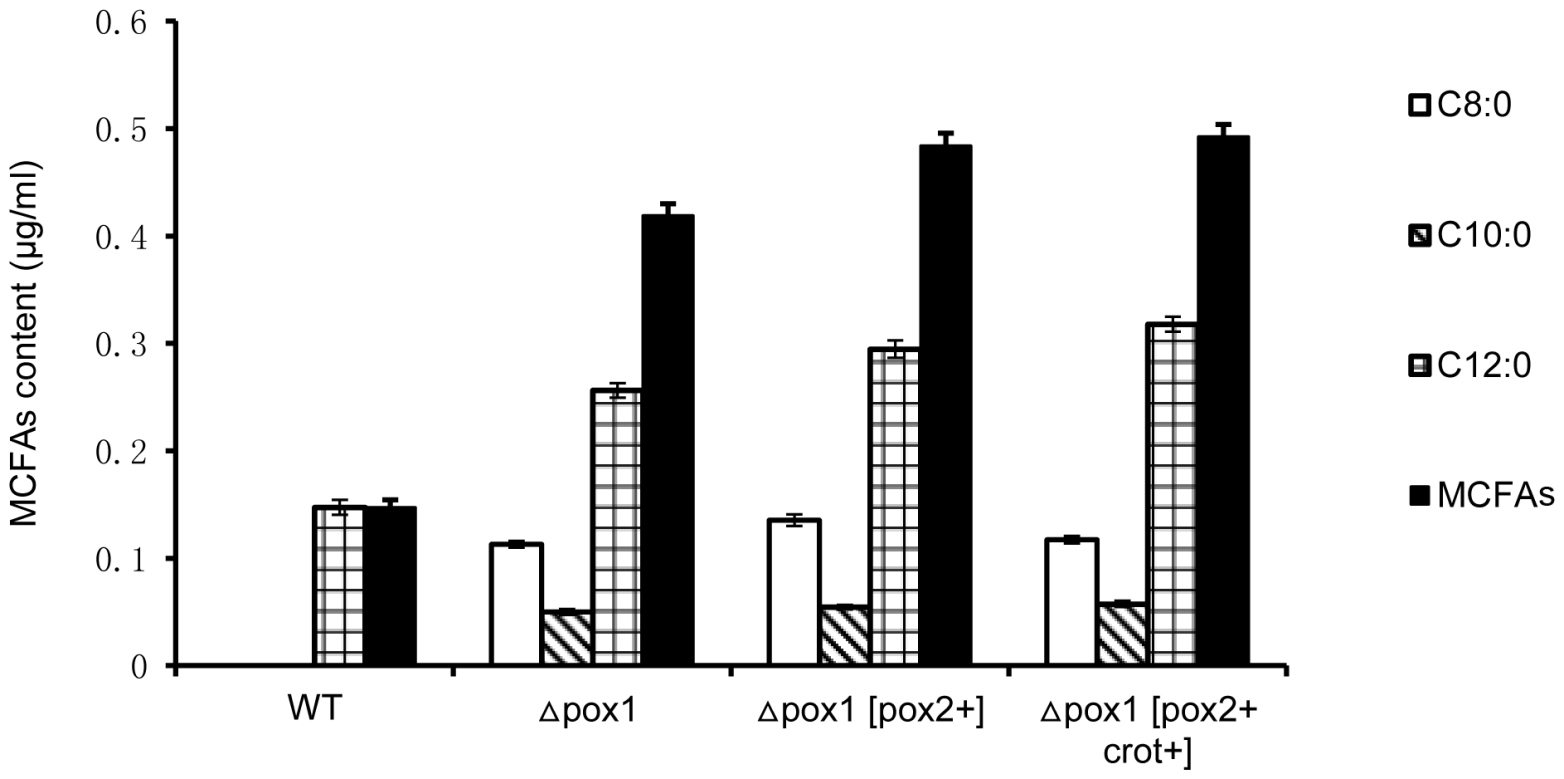
MCFAs content in the culture supernatant of the engineered strains and the WT strain grown for 24_0.5_O_3_ medium. Cells were collected after growth for 24_0.5_O_3_ medium, and the culture supernatant was separated by centrifugation. MCFAs were detected with the SPE method (detailed information referred to in the fatty acids analysis section). Data are the mean values of three independent experiments. The error bar indicates the S.D. C8:0: open bar, C10:0: striped bars, C12:0: gridded bar, MCFAs: solid bar.

Another observed change was an increase in the MCFAs content of the culture supernatants from engineered strains compared to the WT strain. MCFAs (chain length 8 to 12 carbons) are usually a minor constituent of total fatty acids present at a low density in the cell, and C8:0 and C10:0 are not generally detectable in culture supernatant. Because MCFAs can freely traverse the membrane of a cell, when MCFAs were produced, they were secreted into the culture medium. As shown in Figure 6, there was no detectable C8:0 or C10:0 in the WT strain, only C12:0. In contrast, C8:0, C10:0 and C12:0 were all detected in the engineered strains. MCFAs content in the three engineered strains increased 2.84-fold (Δ*pox1*, 0.419 µg/ml), 3.29-fold (Δ*pox1* [*pox2*+], 0.484 µg/ml) and 3.34-fold (Δ*pox1* [*pox2*+, *crot*+], 0.493 µg/ml) compared to the WT strain (0.147 µg/ml).

### Metabolic profiling of control and engineered strains

**Figure 7:**
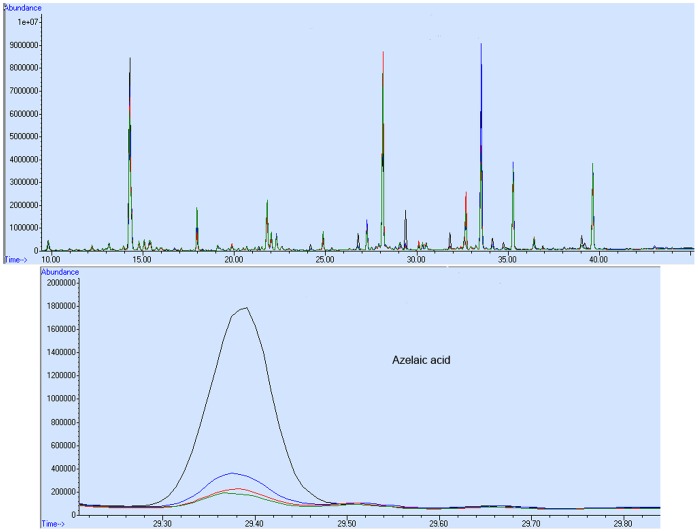
The representative GC-MS spectra for azelaic acid derived from total ion chromatograms. Intracellular metabolites were extracted from the engineered strains and the WT strain, and metabolic profiling was conducted. WT (black), Δ*pox1* (blue), Δ*pox1* [*pox2+*] (red), and Δ*pox1*[*pox2+, crot+*] (green).

**Figure 8:**
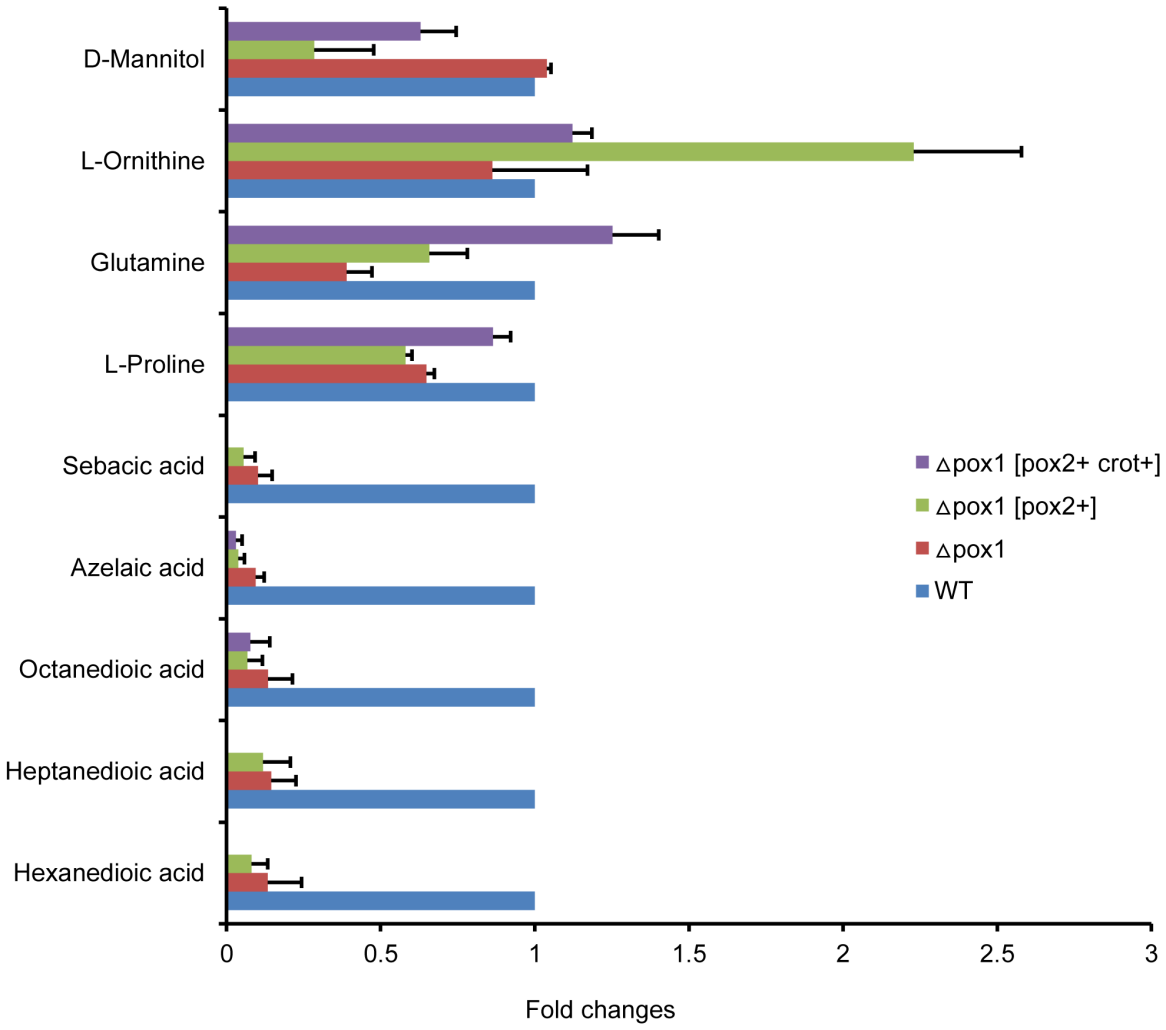
Differential expression levels of intracellular metabolites in the engineered strains compared to the WT strain.

*S. cerevisiae* was modified to maintain the ability to degrade LCFAs but limited ability to degrade MCFAs. To analyze the difference between the engineered strains in the oxidation of MCFAs, GC-MS-based metabolic profiling (Figure 7) was performed. The metabolic responses of the engineered strains were useful in understanding the changes to the fatty acid degradation pathway after gene modification. It was found that there were obvious differences in the cellular metabolite levels between engineered strains and the WT strain, as shown in Figure 8. Fewer dicarboxylic acids were present in the engineered strains compared with the WT strain when cultured in YNBD_0.5_O_3_ medium. This observation is consistent with a previous study [25] reporting that dicarboxylic acids (hexanedioic acid, heptanedioic acid, octanedioic acid, azelaic acid and sebacic acid) are formed when fatty acid oxidation is increased or inhibited. Because the formation of dicarboxylic acids could facilitate the degradation process, there are higher levels of dicarboxylic acids when fatty acid oxidation is increased than when it is inhibited. In the WT strain, dicarboxylic acids were at the highest level, which was due to increased β-oxidation compared to the engineered strains. There were much lower levels of dicarboxylic acids in the Δ*pox1*, Δ*pox1* [*pox2+*] and Δ*pox1* [*pox2+, crot+*] strains, which had negligible β-oxidation of MCFAs. This difference was consistent with the fatty acid metabolism of several strains. With regard to L-proline, glutamine, and L-ornithine, these changes might be related to alterations in the metabolism of the tricarboxylic acid cycle (TCA) cycle. These amino acids are synthesized from intermediates of the TCA. When a strain has a growth defect, the carbon flow toward the TCA cycle becomes restricted, further decreasing the concentration of amino acids related to the TCA cycle. In the strain Δ*pox1* [*pox2+, crot+*], there was no observed growth defect, and the amino acid levels were upregulated relative to normal levels. As for the increase of ornithine in Δ*pox1* [*pox2+*], this may be due to the very low growth rate. Thus, there was a relative excess of nitrogen driving ornithine synthesis.

## Discussion

### Impact of Aox2p and Crot on fatty acids composition in the engineered strains

Our study showed that when oleic acid was used as a co-substrate of glucose, the total fatty acids were increased in engineered strains compared to the WT strain. As shown in Table 1, the total fatty acids from strains Δ*pox1* [*pox2*+] and Δ*pox1* [*pox2*+, *crot*+] were increased compared to the WT strain. In contrast, the total fatty acids of strains Δ*pox1* were not increased. This indicated that the β-oxidation defect had a side effect on fatty acid accumulation other than fatty acid uptake. Modification of the β-oxidation pathway not only affected the total fatty acids content but also changed the composition. There was MCFAs (C8:0, C10:0 and C12:0) accumulation, and C8:0 and C10:0 were secreted into the medium. For the initial gene modification step, we obtained the deletion strain Δ*pox1*. It was observed that there was an increase in C8:0 and C10:0 in Δ*pox1*. From a previous report [26], it had been shown that medium chain acyl-CoAs can be released from the fatty acid synthetase (FAS) complex due to the accumulation of saturated LCFAs during the fermentation conditions. Medium chain acyl-CoAs are hydrolyzed to recycle CoA-SH. MCFAs can diffuse passively through the cytoplasmic membrane. Our findings therefore suggested that medium chain acyl-CoAs might be released from the FAS complex in the Δ*pox1* strain when cultured in oleic acid-containing medium, even under respiratory conditions. In contrast, C8:0 and C10:0 did not accumulate in the WT strain. In Δ*pox1* [*pox2*+], the increase of MCFAs was partly due to the effect of *POX1* deletion and partly due to the expression of *POX2* from *Y. lipolytica*. In the peroxisomes of Δ*pox1* [*pox2*+], only long-chain acyl-CoAs were degraded to medium-chain acyl-CoAs and acetyl-CoA. The degradation product acetyl-CoA was activated to acetyl carnitine and transported out of the peroxisome by carnitine acetyltransferase (Cat2p) [9]. However, there is no transferase in *S. cerevisiae* that is involved in transporting medium chain acyl-CoAs out of the peroxisome and recycling CoA-SH. Although there is a peroxisomal acyl-CoA thioesterase (PTE1) [27] in *S. cerevisiae* to hydrolyze acyl-CoAs of all chain lengths to generate free CoA-SH, this enzyme does not appear to possess sufficient activity to hydrolyze all of the medium chain acyl-CoAs accumulated in peroxisomes in the strain Δ*pox1* [*pox2*+]. Owing to the expression of Aox2p and thioesterase in the peroxisome, there was an increase of MCFAs in Δ*pox1* [*pox2*+]. However, the accumulated medium chain acyl-CoAs led to depletion of free CoA-SH and eventually the termination of the β-oxidation pathway, causing the growth defect. When Crot was expressed in the strain Δ*pox1* [*pox2*+, *crot*+], the carbon flux through β-oxidation became unobstructed because the accumulated medium chain acyl-CoAs could be transported out of the peroxisome, balancing the CoA concentration in peroxisomes. Therefore, the growth rate was recovered to the levels of the WT strain. However, it should be noted that there was not a continuous increase of MCFAs in the strain Δ*pox1* [*pox2*+, *crot*+]. It was assumed that there was an increase of cytosolic medium chain acyl-CoAs in the engineered strains. In future studies, we will detect medium chain acyl-CoAs, as well [28].

### Metabolic engineering of *S. cerevisiae* for MCFAs production

It has been shown that MCFAs accumulation is related to fermentative metabolism in *S. cerevisiae* [26], [29]. During fermentation, fatty acid synthesis is inhibited by the lack of oxygen. As a result, medium chain acyl-CoAs are released from the FAS and hydrolyzed to recycle CoA-SH. Cell growth ceases due to the inhibition of fatty acid synthesis. During our investigation of the role of Aox2p and Crot in fatty acid profile and MCFAs accumulation, we expressed Aox2p and Crot in the Δ*pox1* strain. As expected, the total fatty acids and MCFAs were increased in the engineered strains compared to the WT strain. We showed that in aerobic conditions, MCFAs could be produced in *S. cerevisiae* through modification of the β-oxidation pathway. The metabolic responses in different strains helped us to further investigate the impact of Aox2p and Crot. In the WT strain, there was a high concentration of medium chain dicarboxylic acids detected, which facilitated β-oxidation in the cells [25]. In contrast, the MCFAs oxidation ability decreased dramatically in the engineered strains. A much lower concentration of medium chain dicarboxylic acids were detected. On the contrary, MCFAs were secreted into the culture medium.

In summary, we observed that the production of both intracellular and extracellular MCFAs increased in the engineered stains compared to the WT strain when grown in YNBD_0.5_O_3_. It was demonstrated that modification of β-oxidation was capable of turning low value fatty acid feedstock into higher value fatty acids containing more MCFAs. However, MCFAs remained a minor component of the total fatty acids. The observed increase in total fatty acids indicated that most of the oleic acid in the medium went into the storage pathway instead of degradation pathway. Thus, further investigation is needed to improve the oxidation of LCFAs and the formation of MCFAs. Future studies will aim at the expression of other β-oxidation-related genes and medium chain acyl-CoA hydrolases, such as Acot5 (medium chain acyl-CoA thioesterase) [30]. Meanwhile, endogenously produced lipase can be used [31] to increase the uptake of fatty acid-rich feedstock, such as triacylglycerols (TAG), in the engineered strains. The current work terminated the β-oxidation of yeast in peroxisomes at medium chain acyl-CoAs. It was demonstrated that heterologous expression of β-oxidation enzymes in *S. cerevisiae* system is a promising tool to exploit bioconversion platforms.
